# Adsorptive Removal of Toxic Chromium from Waste-Water Using Wheat Straw and *Eupatorium adenophorum*

**DOI:** 10.1371/journal.pone.0167037

**Published:** 2016-12-02

**Authors:** Dagang Song, Kaiwen Pan, Akash Tariq, Azizullah Azizullah, Feng Sun, Zilong Li, Qinli Xiong

**Affiliations:** 1 Key Laboratory of Mountain Ecological Restoration and Bioresource Utilization & Ecological Restoration Biodiversity Conservation Key Laboratory of Sichuan Province, Chengdu Institute of Biology, Chinese Academy of Sciences, Chengdu, People’s Republic of China; 2 University of Chinese Academy of Sciences, Beijing, People’s Republic of China; 3 Department of Botany, Kohat University of Science and Technology, Kohat, Pakistan; VIT University, INDIA

## Abstract

Environmental pollution with heavy metals is a serious issue worldwide posing threats to humans, animals and plants and to the stability of overall ecosystem. Chromium (Cr) is one of most hazardous heavy metals with a high carcinogenic and recalcitrant nature. Aim of the present study was to select low-cost biosorbent using wheat straw and *Eupatorium adenophorum* through simple carbonization process, capable of removing Cr (VI) efficiently from wastewater. From studied plants a low cost adsorbent was prepared for removing Cr (VI) from aqueous solution following very simple carbonization method excluding activation process. Several factors such as pH, contact time, sorbent dosage and temperature were investigated for attaining ideal condition. For analysis of adsorption equilibrium isotherm data, Langmuir, Freundlich and Temkin models were used while pseudo-first-order, pseudo-second-order, external diffusion and intra-particle diffusion models were used for the analysis of kinetic data. The obtained results revealed that 99.9% of Cr (VI) removal was observed in the solution with a pH of 1.0. Among all the tested models Langmuir model fitted more closely according to the data obtained. Increase in adsorption capacity was observed with increasing temperature revealing endothermic nature of Cr (VI). The maximum Cr (VI) adsorption potential of *E*. *adenophorum* and wheat straw was 89.22 mg per 1 gram adsorbent at 308K. Kinetic data of absorption precisely followed pseudo-second-order model. Present study revealed highest potential of *E*. *adenophorum* and wheat straw for producing low cost adsorbent and to remove Cr (VI) from contaminated water.

## Introduction

Heavy metals contamination is a growing issue affecting living organisms throughout the world [1–2]. Due to rapid industrial development, direct and indirect discharges of heavy metals to the environment through wastewater have tremendously been increased [2]. In industrial wastewater treatment chromium, copper, cadmium, lead, zinc and nickel are considered more toxic and receiving more attention of researchers [3].

Chromium (Cr) is a harmful heavy metal and exists in various oxidative forms; however, the trivalent and hexavalent states are considered more essential in term of environmental pollution point of view [4]. Cr (VI) is considered more harmful and toxic due to its high carcinogenic and resistant properties than Cr (III) [5]. There are varied Cr contamination sources such as electroplating, leather tanning, textile industries, metal finishing, nuclear power plants, and chromate preparation [6]. In many countries, concentration of Cr (VI) is severely restricted and as per Environmental Protection Agency (EPA) guidelines the maximum permitted limit for Cr (VI) in inland surface, potable and industrial wastewaters are 0.1, 0.05, and 0.25 mg L^−1^, respectively. Over ingestion of Cr (VI) beyond the permissible limits can cause severe gastric damage, liver, kidney and lung cancer and other health related complications [7–9]. So there is a dire need of significant management of wastewater contaminated by Cr (VI) before its release.

Different conventional methods such as chemical precipitation, filtration, chemical oxidation and reduction, reverse osmosis, evaporation techniques, electrochemical and ion exchange methods are generally recommended for Cr (VI) removal from the environment [10]. However, these methods are cost effective and generate a variety of secondary pollutants [11]. Adsorption method is known to be the most promising technique and is considered as economically and operationally very effective for removing heavy metals from contaminated water, especially with the application of low-cost and high efficiency adsorbents. Many adsorbents like rice hull [12], henna leaves, chufa corm peels, peel of banana, wheat bran, etc [13–16] been testified very effective for Cr (VI) removal. However, these adsorbents have relatively low adsorption capacity to Cr (VI), therefore, it is necessary to search for highly abundant naturally available, low cost and greater adsorption capacity adsorbent in order to alleviate Cr (VI) ions from contaminated water. Wheat (*Triticum aestivum*) is one of the chief cash crops around the world and approximately 0.6 billion tons of wheat straw produced annually. In addition to fuel and food importance, huge amount of wheat straw is burnt in the field, which not only wastes a potential resource, but also pollutes the environment [17]. Wheat straw is rich in cellulose, hemicellulose, linen and sugars which can be used as complexion chelation with heavy metal ions [18] *E*. *adenophorum* (Crofton weed), is a harmful invasive plant species originated from Central America and currently invaded many countries of Southeast Asia [19]. *E*. *adenophorum* invaded a varied kind of habitats such as road sides, disturbed grasslands, abandoned fields and agricultural fields posing great loss to economics and biodiversity [20]. Utilizing *E*. *adenophorum* as a raw material for adsorbent production could be a useful measure not only for controlling its invasion process but will also utilize it as an economical natural resource. Activated carbon made from *E*. *adenophorum* has already been reported in recent years [21] but the cost is very high and use of chemical drugs during its activation stage also produce new contaminations, which somewhat limit its application as a new adsorbent.

The aim of the present study is to design an adsorbent of low cost wheat straw and *E*. *adenophorum* through simple carbonization process, which can efficiently absorb and remove Cr (VI) from wastewater. The adsorbent prepared from wheat straw and *E*. *adenophorum* was studied by optimized technological parameters and effects of different parameters such as adsorbent dosage, contact time, pH and solution temperature were investigated using a batch process. Absorption capacity rate and the mechanism of Cr adsorption were measured with the help of various equilibrium isotherm and kinetic models. This study would provide baseline information to researchers about using the abundantly available low-cost adsorbent that could be helpful in reducing the problem of Cr (IV) polluted water across the world.

## Materials and Methods

### Ethics statement

In present investigation *E*. *adenophorum* and wheat straw were collected from Xichang city, Sichuan Province, southwestern China. No specific permission was required for the collection of these species because of its high abundance in the area, not endangered or protected species and not under any authority responsible for national park or protected areas of land or sea. Therefore, there is no restriction or need of permission for harvesting these species for research activities in Xichang city.

### Preparation of the adsorbent

The raw materials (stems) were dried at 378 K then ground in a mill and finally passed through (-35, +60) meshes. *E*. *adenophorum* stem and wheat straw powders were mixed in different combinations as follows: (a) *E*. *adenophorum* (50%) + wheat straw (50%), (b) *E*. *adenophorum* (33%) + wheat straw (67%), (c) *E*. *adenophorum* (100%) + wheat straw (0%), (d) *E*. *adenophorum* (25%) + wheat straw (75%), (e) *E*. *adenophorum* (67%) + wheat straw (33%), and (f) *E*. *adenophorum* (0%) + wheat straw (100%). These combinations were carbonized at different temperatures (i.e., 673, 723, 773 and 823 K) for different times (i.e., 15, 30, 45 and 60 min) and these samples were then used as adsorbent.

### Reagents and Equipments

For preparing Cr (VI) (45 mg/L) stock solution, 0.1415 ± 0.0001g of the analytical grade potassium dichromate (K_2_Cr_2_O_7_) (Chengdu, China) was dissolved in deionized water. The stock solution was further diluted for the preparation of working solutions. Digital pH (PHS-25, Shanghai, China) meter was used for measuring pH. For samples igitation electric thermostatic reciprocating shaker (SHZ-88, Jintan, China) was used. The content of chromium in standard and treated solutions was determined using a visible spectrophotometer (Model 722N, Shanghai, China). *E*. *adenophorum and* wheat were carbonized with the help of an electric furnace (Model TMF-4-13, Shanghai, China). In aqueous solution particular concentration of Cr (VI) was determined with the help of spectrophotometer using 1, 5-Diphenylcarbazide reagent (GB 7467–87, China), which specifically measures Cr (VI). Colorimetric method using diphenylcarbazide reagent (GB7466-87, China) was used for the measurement of total Cr.

### Batch adsorption studies

For the investigation of solution temperature, contact time, adsorbent dosage, and pH batch studies were carried out. Designated adsorbents were added into 150 ml conical flask with a stopper, containing 100 ml of test solution and then carried out the batch adsorption at the desired contact time, pH and adsorbent dose. Solutions with varied initial concentrations of Cr (VI) were prepared using standard stock solution dilution of 45 mg/L Cr (VI). pH adjustment was done with 0.1 mol/L NaOH and 0.1 mol/L HCl. After each batch, the solution was filtered and the filtrate was analyzed for remnant Cr (VI) concentration in the sample.

Prepared biosorbent (0.3g) added to 100 mL of Cr (VI) solution (45 mg/L) in a conical flask with a stopper. The conical flask was shaken in electric thermostatic reciprocating shaker for 3 h at 298 K with the frequency of 150rpm. The removal percentage of Cr (VI) was calculated based on the measured value of *C*_*i*_ and *C*_*e*_ concentrations. The removal percentage of Cr (VI) by the adsorbent at different periods and temperatures were measured and calculated.

The removal percentage of Cr (VI) was calculated as follows:

$\%R=(1-\frac{C}{C_{0}})\times 100\%$ Where %*R* indicates the removal percentage of metals by biosorbent, *C* stands for final concentration and *C*_*0*_ is the initial concentration of metal in the solution (mg/L).

The adsorbed amount of Cr (VI) in the equilibrium was determined with the help of following equation;

$$q_{e}=\frac{V(C_{i}-C_{e})}{m}$$

Where q_e_ is Cr (VI) quantity adsorbed by per gram of adsorbent (mg/g); *V* indicates volume of the solution (L), *C*_*i*_ and *C*_*e*_ (mg/L) are the initial and equilibrium metal concentrations respectively, and *m* is the weight (g) of adsorbent.

### Adsorption kinetic studies

The kinetic tests were also carried out with same technique as in the equilibrium experiments. In the adsorption kinetic study, pseudo-first-order, pseudo-second-order, intra-particle diffusion and external film diffusion models were followed in order to estimate the adsorption rates and possible reaction mechanisms. The solutions were shaken for 3 h at a frequency of 150 r/min. During the course of experiment, samples were periodically taken from each solution at fixed time intervals (10, 20, 30, 40, 50 and 60 mins).

The pseudo-first-order kinetic model was expressed as:

$$\log(C_{e}-q_{e})=\log C_{e}-\frac{k_{1}t}{2.303}$$

The pseudo-second-order kinetic model was described as:

$$\frac{t}{q_{t}}=\frac{1}{k_{2}q^{2}{}_{e}}+\frac{t}{q_{e}}$$

Where *q*_*t*_ and *q*_*e*_ are Cr (VI) quantity adsorbed (mg/g) at specific adsorption time and at equilibrium, respectively. *k*_*1*_, *k*_*2*_ are pseudo-first and second-order adsorption rate constants (per minute) respectively.

External film diffusion [22] and intra-particle diffusion [23] models were followed for the investigation of rate controlling step in the process of adsorption. These two equations are defined as follows:

$$q_{t}=k_{i}t^{0.5}+c$$

$$\ln(1-\frac{q_{t}}{q_{e}})=-k_{fd}t$$

Where *k*_*i*_ (mg/g min^0.5^) and *k*_*fd*_ (1/min) represents intra-particle diffusion and liquid film diffusion models rate constants, respectively.

Cr (VI) adsorption kinetics was measured from the curves of time against *q*_*e*_ (mg/g). Linearized form of pseudo-first-order model for Cr (VI) ions adsorption onto wheat straw and *E*. *adenophorum* at various adsorption times (min) at 298 K. Conformation between model predicted values and experimental results were stated by correlation coefficient (R^2^). Adsorption density *q*_e_ and rate constant k_1_ or k_2_ values were attained from the intercepts and slopes of the plot of log(*C*_*e*_−*q*_*e*_) against *t*, respectively (Table 1). If intra-particle diffusion has major role in term of controlling kinetics of adsorption process, the plot of *q*_*t*_ versus *t*^0.5^ produces straight line passing through the origin, and the slope gives the rate constant, *k*_*i*_. Similarly, if external film diffusion equation is appropriate, the plot of ln(1-*q*_*t*_/*q*_*e*_) against *t* should give a straight line with zero intercept, from which *k*_*fd*_ can be measured from the slope of the plot (Table 1).

**Table 1:** Kinetic constant parameters obtained for Cr (VI) adsorption on biosorbent.

pseudo–first- order				pseudo–second-order		
q_e,exp_/ (mg/g)	q_e,cal1_(mg/g)	k_1_(10^−3^)(min^-1^)	R^2^	q_e,cal2_(mg/g)	k_2_(10^−3^)(min^-1^)	R^2^
17.7	17.579	4.14	0.9529	17.844	0.06128	0.9998

### Adsorption isotherms experiments

Isothermal models like Freundlich, Langmuir and Temkin were considered for describing Cr (VI) uptake carbonized plant materials. The Temkin isotherm model suggested decrease in adsorption energy linearly with surface coverage due to interaction between adsorbent and adsorbate. Equilibrium isotherm was calculated at a temperature of 298, 303, and 308 K and agitation speed of 150 rpm. In 100 ml of solution total 0.25 g adsorbent was added with different concentrations of Cr (VI), i.e.0, 50, 75,100, 150, 200, 300 and 400 mg/ L. Solution pH was adjusted to 1.0 and the model parameters were determined from the following equations:

$$Langmuir\;isotherm\;q_{e}=\frac{q_{m}bC_{e}}{1+bC_{e}}$$

$$Freundlich\;isotherm\;\ln q_{e}=\ln K_{f}+\frac{1}{n}C_{e}$$

Where *q*_*e*_ is Cr (VI) quantity adsorbed by per gram of adsorbent (mg/g); *q*_*m*_ stands for highest adsorption capacity (mg/g); b indicates equilibrium constants; *C*_*e*_ is Cr (VI) (mg/L) equilibrium concentration; *K*_f_ is Freundlich isotherm constants; *n* is Freundlich constant; *n* were determined using ln*q*_*e*_ versus *C*_*e*_ intercept and the slope of a plot of ln*q*_*e*_ versus ln *C*_*e*_ (Table 2). If 1/*n* value were found less than 1, it designated a favorable adsorption.

**Table 2:** Parameters of different isotherms of adsorption.

T/K	Langmuir			Freundlich			Temkin isotherm		
	*Q*_*m*_ (mg/g)	*K*_*c*_	R^2^	*K*_*f*_	1/n	R^2^	*b*_*T*_ (KJ/mol)	*K*_*T*_ (L/gm)	R^2^
298	56.91	0.069	0.9202	27.98	0.102	0.8783	0.7266	6.735	0.7251
303	64.39	0.591	0.9751	43.479	0.09	0.6967	0.5049	6.847	0.5861
308	88.57	0.801	0.9974	47.197	0.131	0.893	0.3976	4.679	0.9497

Also, the linear form of the Temkin isotherm model was described as follows [24]:

$$q_{e}=\frac{RT}{b_{T}}\ln K_{T}+\frac{RT}{b_{T}}\ln C_{e}$$

Where *b*_*T*_ is the Temkin constant related to the heat of sorption (J/mol), *K*_*T*_ represents equilibrium constant (L/g) binding, *T* indicates absolute temperature (K), and *R* is gas constant (8.314 × 10^−3^ kJ/mol K^-1^). The Temkin constants were calculated from the intercept and slope of a linear plot of *q*_*e*_ vs ln*C*_*0*_ (Table 2).

## Results and Discussion

### Optimizing technological parameters for the preparation of adsorbent

**Fig 1:**
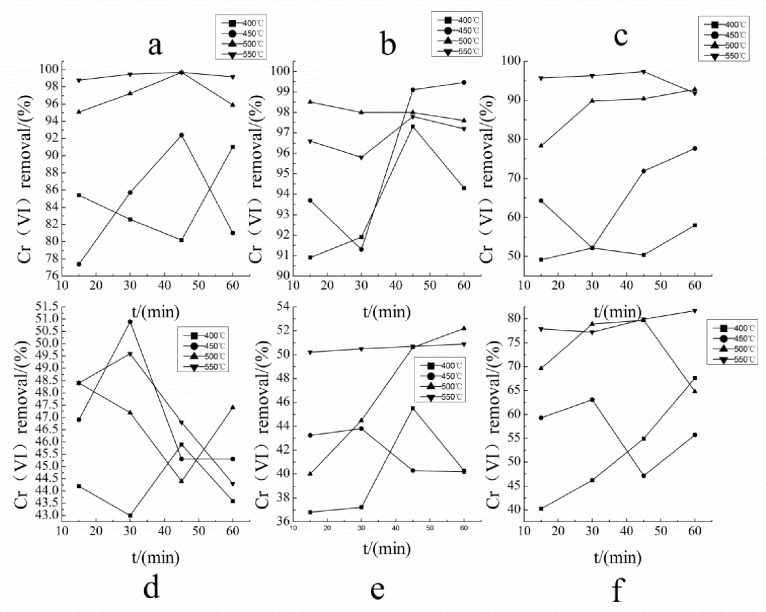
Optimizing technological parameters, initial Cr (VI) concentration 45 mg/L, pH 1.0.

The optimal technological parameters were selected according to the optimum removal percentage of Cr (VI). The carbonizations parameters corresponded to the highest removal ratio of each mixed materials are shown in Fig 1. The results showed that at a carbonization temperature of 773 K, carbonization time of 45 min, with the combination of materials of *E*. *adenophorum* (50%) and wheat straw (50%), the percentage removal of Cr (VI) was 99.7% (Fig 1A). All the experiments had three replications and mean value was used.

### Effect of pH on Cr (VI) adsorption

**Fig 2:**
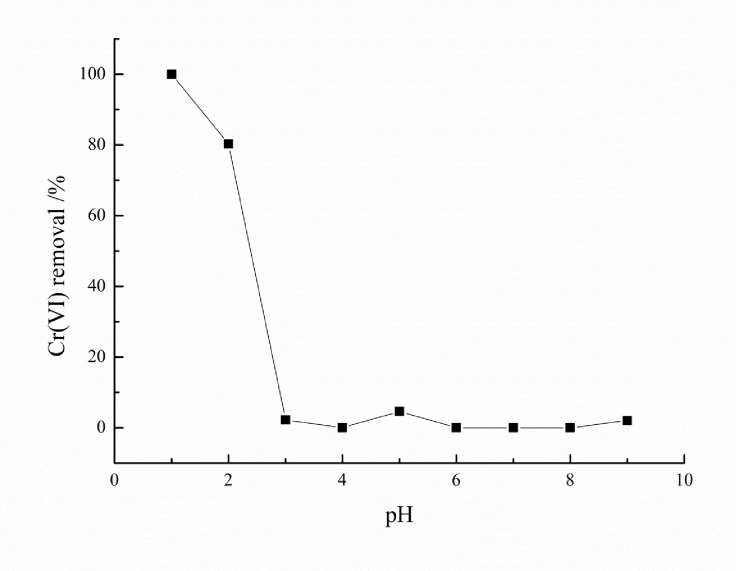
Effect of pH on Cr (VI) adsorption at initial Cr (VI) concentration of 45 mg/L, adsorbent dosage of 0.3 g/100mL, time 3 h and temperature 298 K

The obtained results revealed that pH strongly affected the % adsorption (Fig 2). The Cr (VI) removal percentage was found to decrease with an increase in pH. The removal efficiency maintained at 5% with little fluctuations within the pH range of 3 to 9. The Cr (VI) removal percentage reached to the maximum of 99.98% at a pH of 1. Previous studies on adsorption of heavy metals suggested pH is an essential factor affecting the efficiency of adsorption process [25], since it interferes with the solid–solution interface. It affects biomass active site charges and also influences behavior of metals in solution [26]. According to the solution pH, Cr (VI) mainly exists as: chromate (CrO_4_^2-^), chromic acid (H_2_CrO_4_) hydrogen chromate (HCrO_4_^-^) or dichromate (Cr_2_O_7_^2-^) [27–28]. Change in Cr (VI) adsorption with changing pH might be due to the fact that at low pH of solution the adsorbent can easily protonate and generate positive net charge. Thus, increase adsorption of Cr (VI) attained with decreased pH of the solution due to enhancement of electrostatic attraction [29]. A lot of positive charges (-COOH_2_^+^, -NH_4_^+^, -OH_2_^+^) were present and covered the entire adsorbent surface resulted in increase of contact area between Cr (VI) and adsorbent at pH between 1 and 2. As a result, more Cr (VI) was absorbed by the carbonized wheat straw and E. adenophorum. As pH increased, abundance of OH^−^ ions on the adsorbent surface caused repulsive force between adsorbent and adsorbate in the solid–liquid phase, which resulted in a reduction of the adsorption [30]. Also, at pH 1, Cr mostly exists as HCrO_4_^-^, but with an increase in pH, HCrO_4_^-^ shuffles to Cr_2_O_7_^2-^ and other forms such as CrO_4_^2-^. Finally, as the pH is increased above 7.0, CrO_4_^2−^ would be the primary form [31]. These results clearly revealed that HCrO_4_^-^ was preferentially adsorbed onto the biomass, thus inconsistent with previous findings [23, 32]. It can be speculated that HCrO_4_^-^ preferably adsorbed on the adsorbent, however, the discrepancy observed may be due to the fact that the materials of the adsorbent in the present study were different from the earlier studies.

### Effect of adsorbent dosage on Cr (VI) adsorption

**Fig 3:**
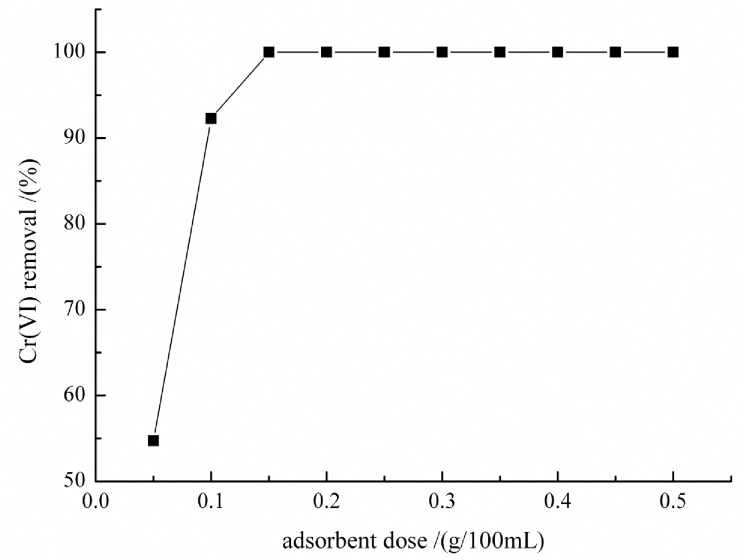
Effect of adsorbent dosage on Cr (VI) adsorption at initial Cr (VI) concentration 45 mg/L, pH 1.0, time 3 h and temperature 298 K

Increase in adsorbent amount from 0 to 0.15 g/100mL, Cr (VI) removal percentage increased rapidly and reached 99.9% at the dosage of 0.15 g/100mL (Fig 3). At a dosage of 0.25 g/100mL, the removal efficiency gradually reached adsorption equilibrium and there was no further increase in the adsorption of Cr with further increase in adsorbent dosage. Similar findings have also been reported in earlier studies [33–35].

Adsorbent dosage is an important factor for the determination of adsorption potential of an adsorbent at a given initial concentration [36]. The variation in adsorption capacities at different dosage of adsorbents could be mainly attributed to the available sites of adsorption. As the adsorbent dosage increased, more active sites and surface area of the adsorbent became available for the adsorption. When the adsorption reached an equilibrium, increase in adsorbent dosage had no effect on the adsorbate uptake because it added unavailable sites [37]. Furthermore, although adsorbent dosage increased the interference of the adsorbent surface among the active groups, the equilibrium concentration of Cr (VI) was lower; because the driving force was too low for adsorb to diffuse into the adsorbent surface to be adsorbed. These findings are in accordance with previous studies [38].

### Effect of the contact time and conformation changes of Cr (VI)

**Fig 4:**
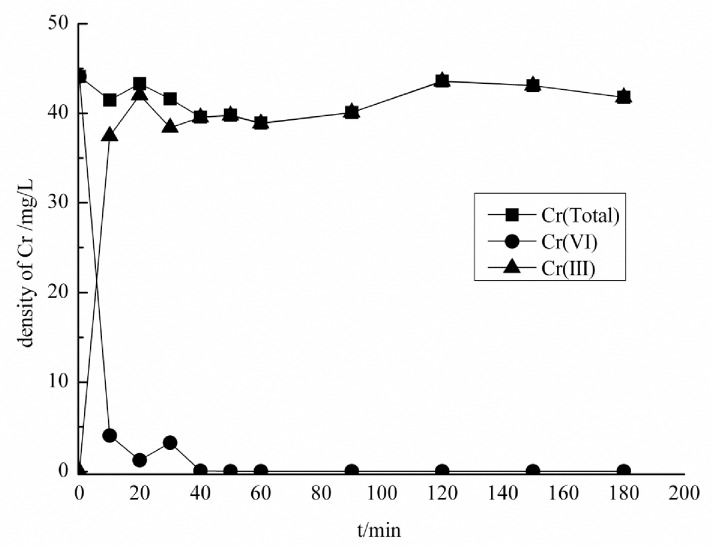
Effect of contact time on Cr (VI) adsorption, initial Cr (VI) concentration 45 mg/L, pH 1.0, adsorbent dosage 0.25 g/100mL and temperature 298 K

During 3 h adsorption process, the samples were withdrawn at 0, 10, 20, 30, 40, 50, 60, 90, 120, 150 180 mins intervals. The results of total chromium and Cr (VI) measured showed that Cr (VI) in the solutions decreased sharply with the extension of adsorption time, and reached adsorption equilibrium after 50 mins and was completely removed in the aqueous solution (Fig 4).

The final concentration of Cr (III) persisted at 38.4mg/L after the complete removal of Cr (VI), suggesting that biomass adsorbed 6.6 mg/L of total Cr. As described in Fig 4 during first 20 min of adsorption process, total Cr first increased and then gradually decreased, Cr (VI) also decreased while Cr (III) generated. During 20 to 30 min, the decrease of total Cr and Cr (III) was almost equal while slight increase in Cr (VI) was observed. Moreover, from 30 to 50 min, the decrease of total Cr was less than that of Cr (VI), and Cr (III) in the solution found increase. After 50 min, the concentration of Cr (III), Cr (VI), and total Cr tended to be constant in the solution. The concentration of Cr in aqueous solution revealed that the implication of Cr (VI) removal mechanism of the adsorbent was its reduction into Cr (III). This mechanism consisted of three steps: (1) the binding of anionic Cr (VI) ions with the positively charged groups present on the biomass surface; (2) the reduction of Cr (VI) to Cr (III) by adjacent electron-donor groups; (3) the release of the Cr (III) ions into the aqueous phase due to electronic repulsion between the positively charged groups and the Cr (III) ions, or the formation of complexes of the Cr (III) with adjacent groups capable of Cr-binding. Present results are in line with previous studies conducted by Park et al. and Deng et al [39, 40]. These findings suggested that almost complete Cr (VI) was reduced spontaneously to Cr (III) as soon it was in contact with carbonized *E*. *adenophorum* and wheat straw comprising high quantity of reducing C and Cr (VI) has a high redox potential (above + 1.3 V under standard condition) [38, 41–42]. Extension in time of absorption could cost much for engineering application therefore, 50 mins is appropriate time of contact for equilibrium adsorption of Cr (VI) by wheat straw and *E*. *adenophorum*.

### Adsorption kinetic studies

**Fig 5:**
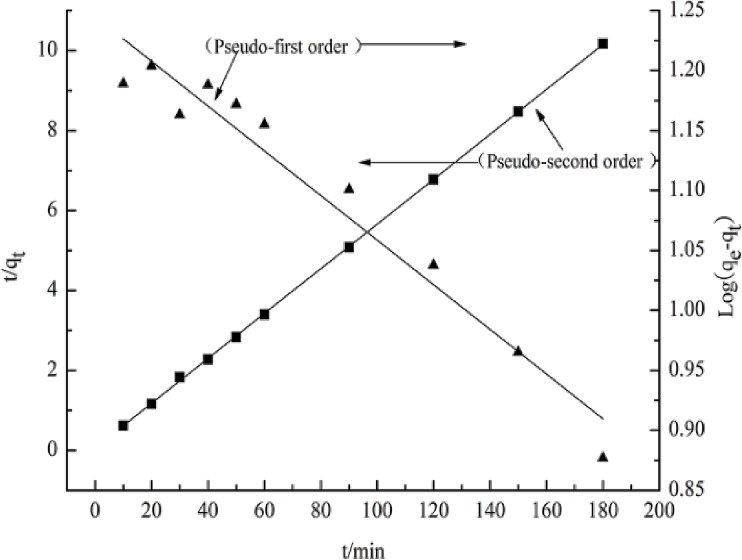
Pseudo–first-order and pseudo–second-order kinetics plots for the adsorption of Cr (VI) onto biosorbent, initial Cr (VI) concentration 45 mg/L, pH 1.0, adsorbent dosage 0.25 g/100mL and temperature 298 K

Table 1 shows values of k_1_, k_2_, measured adsorptive capacity, q_e_, cal and correlation coefficients, R^2^ obtained from the plots for adsorption of Cr (VI) on the wheat straw and *E*. *adenophorum*. Table 1 shows that the R^2^ values of pseudo-second-order model were nearly unity (0.999) for Cr (VI), indicating that the adsorption of Cr (VI) onto wheat straw and *E*. *adenophorum* fitted well with this model (Fig 5). Additionally, data obtained for equilibrium adsorption capacities (q_e, cal_) (17.84mg/g) from pseudo-second-order kinetic model fitted with the experimental values (q_e, exp_) (17.7mg/g). Therefore, this model well explained the adsorption kinetics, suggesting Cr (VI) process is a chemical behavior and in line with previous study of Chen et al. [43].

The plots of ln(1-*q*_*t*_/*q*_*e*_) against t was linear and also with low intercepts (Table 1). Although, the intercepts values were smaller and not absolutely zero but it indicates that in adsorption process external film diffusion had major role [44]. Values of correlation coefficients and Intra-particle diffusion rate constant *k*_*i*_ are given in Table 3. According to the present findings diffusion of Cr (VI) into the pores of adsorbent is not due to adsorption and kinetic studies indicates that diffusion into adsorbent pores is not a leading cause for monitoring adsorption process mechanisms.

**Table 3:** The intra-particle and liquid film diffusion rate constants for the Cr (VI) adsorption.

Intra-particle diffusion			Liquid film diffusion		
*k*_*i*_(mg/(gmin^0.5^))×10	c(mg/g)	R^2^	*k*_*fd*_(1/min) ×10	Intercept	R^2^
8.58	9.50449	0.4432	0.2125	2.98996	0.4223

### Adsorption isotherms experiments

**Fig 6:**
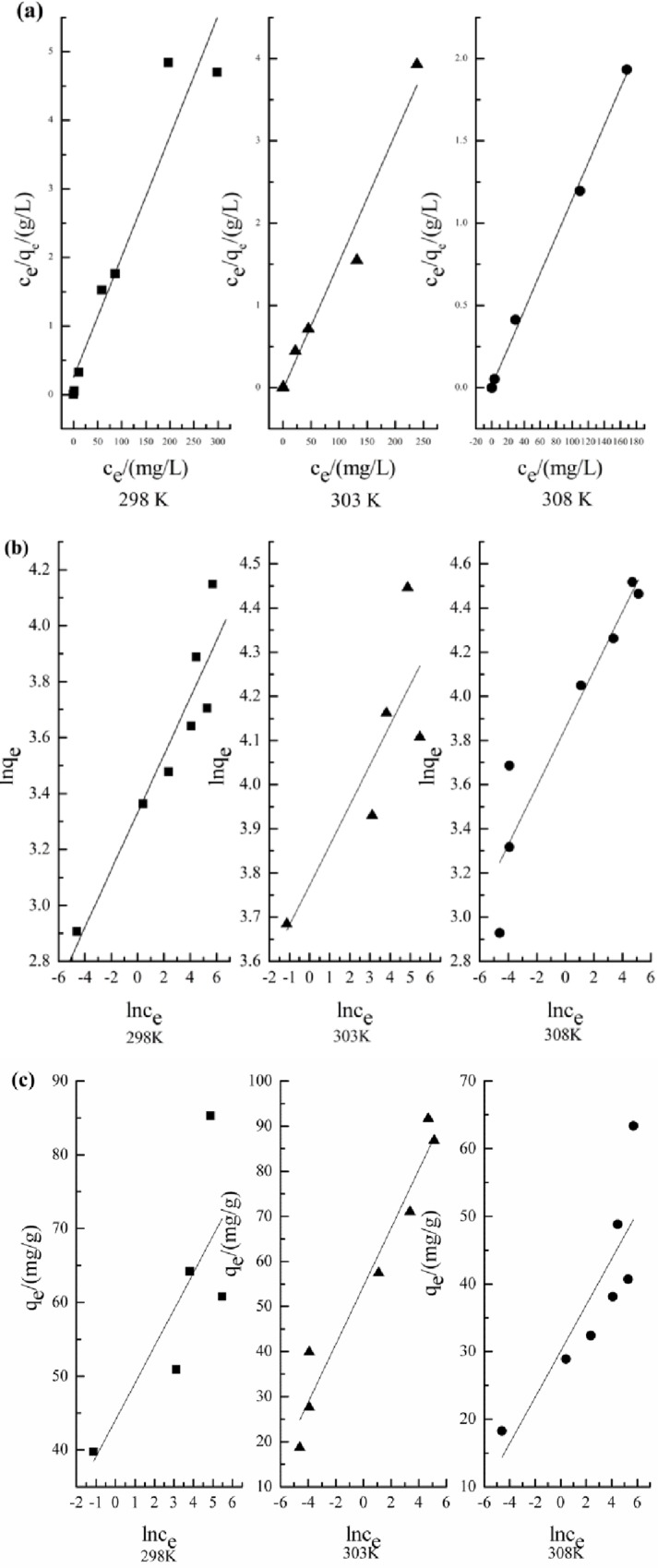
**Adsorption isotherms of Cr (VI) at different temperature (Langmuir (a), Freundlich (b), and Temkin isotherm (c) models)**

Temperature effect on Cr (VI) biosorption was mainly studied at 298K, 303K, and 308K at pH 1 with altered initial Cr (VI) concentration. Cr (VI) removal rate was improved with increase in temperature of the solution. Slope and intercept were used for the estimation of isotherm constants Fig 6(A) (Langmuir isotherm), Fig 6(B) (Freundlich isotherm) and Fig 6(C) (Temkin isotherm) and are presented in Table 1. Coefficient of determination (i.e. R^2^) value was recorded in range of 0.92 to 0.99 and comparatively higher for Langmuir isotherm than for the Temkin isotherm and Freundlich isotherm. High correlation was observed with Langmuir model within the studied temperature range. Present findings suggested that adsorption process was well representing with Langmuir equation. Therefore, development of monolayer coverage of adsorbate over surface of adsorbent was put forward. Value of maximum potential of sorbent (*q*_*m*_) was measured from Langmuir plots. Progressive increase from 56.91 to 88.57 mg/g was observed in maximum capacity of wheat straw and *E*. *adenophorum* for Cr (VI) with elevated temperature, suggesting endothermic adsorption process.

The adsorbents potential for removing Cr (VI) from wastewater in present study is compared with reported values from literature of different adsorbents (Table 4). Although, previous documented studies are the result of different experimental conditions, however, still it could be taken as useful criteria for comparison of adsorption capacities of varied adsorbents. Literature review showed wheat straw and *E*. *adenophorum* have highest adsorption potential for Cr (VI).

**Table 4:** Comparison of maximum sorption capacities of various adsorbents for Cr (VI).

Adsorbents	Temp. (K)	q_m_ (mg/g)	Reference
Sawdust	303	41.5	[45]
Wheat bran	303	40.8	[46]
Rice bran	303	58.9	[46]
Alligator weed	303	82.57	[47]
Parthenium weed	293	24.5	[48]
Silica-based adsorbent	298	68	[30]
Coconut shell carbon	298	20	[49]
*E*. *adenophorum* and buckwheat	303	55.19	[17]
Rice husk	298	10.4	[50]
Carbonized pineapple leaves	293	18.77	[51]
Cactus	303	7.082	[52]
Wheat straw and *E*. *adenophorum*	308	88.57	Present Research

## Conclusions

The maximum adsorption capacity of the studied adsorbents for Cr was observed at a pH of 1. Maximum uptake of Cr was attained at 0.25 g/100mL adsorbent dosage and it can be considered as optimum dosage level of adsorbent under the specified conditions. For the adsorption of Cr from the aqueous solution stable time was 50 min. Experimental results were more fitted to the data attained from Langmuir adsorption isotherm model at different level of temperatures. Pseudo-second-order kinetic model was best fitted for Cr (VI) adsorption and was mutually controlled by film diffusion and intra-particle diffusion for wheat straw and *E*. *adenophorum* adsorbents. Present research confirms that carbonized wheat straw and *E*. *adenophorum* were reliable low cost adsorbent. Considering the economic aspects of wastewater treatment and reducing water pollution, these materials can be utilized as promising and reliable biosorbent for effective removal of Cr (VI) from wastewater.
